# Supplementary material for: The Design of Health Promoting Outdoor Environments for People with Young-Onset Dementia—A Study from a Rehabilitation Garden
Source: Int J Environ Res Public Health. 2024 Aug 9;21(8):1047. doi: 10.3390/ijerph21081047 (PMC11353659; doi:10.3390/ijerph21081047)
Supplement: Supplementary file 1 [file ijerph-21-01047-s001.zip › ijerph-3060199-supplementary.pdf]

Environmental qualities of the Quality Evaluation Tool found in *Bengtsson & Grahn 2014* (highlighted in dark blue) [69] and *Bengtsson et al. 2024* (highlighted in light blue) [33].

Due to developments and changes made by QET over the years, some differences between the two versions may be detected (such as words, descriptions, names and order of qualities), but as both have been used as the basis for the present study both are presented here.

In cases where the quality names from the original article (Bengtsson & Grahn 2014) are different from later versions, the name is indicated in dark blue and in parentheses.

Table S1. Environmental qualities in group A of the QET (Bengtsson & Grahn 2014; Bengtsson et al. 2024)

GROUP A. Six environmental qualities "to be comfortable in the outdoor environment"/ Environmental qualities for people to be comfortable in the green area	
QUALITY	DESCRIPTION
A1. Closeness and easy access	The outdoor environment is physically close to, visible and easily accessible from those parts of the indoor environment where users spend time. Technical properties, e.g. locking devices, doors and thresholds, support both getting outdoors and getting back inside.<br><br>The area is close at hand for users. It is easy to spot and easy to access.
A2. Entrance and enclosure	The enclosure of the outdoor environment corresponds to the degree of safety and security needed by the users. However, the garden must not feel confined. Consider whether gates should be disguised, e.g. as part of the fence, to protect users with cognitive difficulties who may be prone to wandering outside the garden. On the other hand, a deliberate design of the entrance to the garden, creating a distinction between the outer world of everyday life and struggle, and the garden as a safe place where you do not have to keep up appearances, is beneficial to sensitive users.<br><br>The entrances are clear and welcoming. The enclosure of the outdoor environment (hedges, fences, etc.) corresponds to the level of protection that is needed by the users.
A3. Safety and security	(a) The outdoor environment is safe and secure to use without risking any physical unpleasantness, e.g. the risk of falling or sliding, of toxic plants and of falling into water. Ground covers are accessible with regard to their width, surface, gradient and edges. Distances between benches and the availability of handrails fit the users' needs<br><br>(b) The outdoor environment is safe and secure to use without risking any psychological unpleasantness, e.g. the risk of intrusion or of unwillingly being viewed by outsiders. Con- sider the risk of garden users possibly intruding on the privacy of those situated indoors and vice versa. Take into consideration that ambiguous design elements are more likely to cause stressful reactions to fragile

	and vulnerable people than to healthy individuals (Ulrich, 1999). In a healthcare garden, sensitive users often perceive soft shapes and soft colours, e.g. green, lilac, blue and white, as comfortable and soothing, whereas hard angular shapes and intense colours, e.g. red, orange and yellow, are too demanding to them. Furthermore, sounds of nature, e.g. wind and water, are preferred, whereas manmade sounds and sounds of city life are perceived as disturbing. Accordingly, shapes, colour schemes and sounds should be in line with the gradient of challenge, thus placing more challenging features in places where users can choose to go or not to go.<br><br>a) Risks of physical discomfort are very small, such as the risk of falling or slipping, risks of poisonous plants, etc. Ground coverings are accessible in terms of width, surfaces, edges, and slopes. The distance between benches suits users and there are handrails to hold where needed.<br><br>(b) The risks of psychological discomfort in the outdoor environment are very small; the outdoor environment is appealing, without intrusive elements that can be interpreted negatively.
A4. Familiarity	The outdoor environment appears to be a natural part of the healthcare setting. It is easy to familiarize oneself with the outdoor environment. Different parts of the outdoors are perceived to be connected in a whole (i.e., to have high coherence), which is important to familiarity. Garden features, plants and activities are familiar to users and help them feel at home. People in the environment are familiar to the users.<br><br>The green area appears to be a natural part of the environment as a whole and the various elements and activities that the green area offers are easy to comprehend.
A5. Orientation and wayfinding	The distribution and design of paths, places, landmarks, nodes and edges are distinct and aid in understanding and orientation. For instance, it is important for users with orientation difficulties to have paths without dead ends and to have a variety of distinct places along these paths that offer different experiences and activities. There should be major landmarks, such as the doorway back into the building, that can be seen from everywhere in the garden. Boundaries between private places and public places need to be clear. The balance of complexity and unity support the user's ability to orientate in the environment.<br><br>The design of paths, places, landmarks, nodes, and edges is clear and helps users to understand and to be able to orient themselves in the outdoor environment. For people with difficulties in orienting themselves, it is important, for example, that paths do not lead to dead ends and that a variety of places along the paths provide opportunities for different experiences and activities.
A6. Different options in different kinds of weather	Paths and places should offer variation in terms of sun, shade, protection from the wind and shelter from the rain

	Paths and places offer variation in terms of sun, shade, protection from the wind, and shelter from the rain.

Table S2. Environmental qualities in group B of the QET (Bengtsson & Grahn 2014; Bengtsson et al. 2024)

GROUP B. 13 environmental qualities of "access to nature and surrounding life"/ Environmental qualities for stimulation and positive impressions	
QUALITY	DESCRIPTION
B1. Contact with surrounding life	It is possible to engage in the life going on in the surroundings, e.g. things that move and change, pets, people, traffic and city/community life. Consider the possibility of viewing the surroundings from different places in the garden and whether paths should connect the garden with the surroundings.<br><br>The space offers possibilities to take part in life, for example, to experience people, animals, and movement.
B2. Social opportunities	There are places for amusement and pleasure where you can meet and look at people. There are plants and things to discuss. There are areas with outdoor tables and chairs for informal meetings. There are possibilities to socialize in different ways, e.g. places where many people can gather, places for users and visitors to be together by themselves, and places that offer the possibility to interact with people from outside the healthcare setting.<br><br>There are opportunities for entertainment as well as places where it is possible to meet other people. In these places, there are plants and other things to talk about. There are seating options that make it easy to meet and socialize outdoors.
B3. Joyful and meaningful activities	Activities provided in the garden correspond to the user's wishes and needs and are in line with the gradient of challenge. There are areas for stationary activities (e.g., relaxing, drinking coffee and reading), social activities, physical activities, therapy activities and garden activities. There are different walking routes: those for contemplative use as well

	as for exercise. There are possibilities for children to visit the garden to play and interact with the environment.<br><br>There are places for sedentary activities (e.g., relaxing, drinking coffee, reading), social activities, and physical activities. There are walking paths that can be used for exercise as well as for leisurely walks. There are opportunities for children to play and interact with the outdoor environment.
B4. Culture and connection to past times	There are areas that offer fascination with human culture, and that show signs of people's values and toil. There are elements that stimulate memory, such as a clothesline, a hand pump or a barbecue. Design and content give the environment its own special character and meaning and are something to be proud of.<br><br>There are places in the outdoor environment that provide an opportunity to be fascinated by human culture and values. There are objects that stimulate memory. Plants and elements of the outdoor environment give the place its own character and meaning.
B5. Openness (Prospect)	There are inviting green open spaces and views of well-managed nature, greenery and plants.<br><br>There are inviting open green spaces overlooking nature and plants.
B6. Species richness and variety (Rich in species)	There are areas with a variety of species of animals and plants that offer diverse expressions of life.<br><br>There are areas with species richness in terms of plants and/or animals that give varying expressions of life.
B7. Sensory experiences of nature (Sensual pleasures of nature)	There are opportunities to see, feel, hear, smell and taste the gifts of nature, e.g. trees, plants, flowers, fruits, animals and insects. There are opportunities to experience natural elements such as the sun, sky, wind, water, dawn and dusk.

	There is the opportunity to see, feel, hear, smell and taste what nature offers, such as trees, plants, flowers, fruits, animals and insects. There is an opportunity for nature experiences of sun, sky, wind, water, sunrise and sunset.
B8. Seasons changing in nature	There are opportunities to follow the seasons changing as reflected in plants, experiences and activities outdoors, thus offering temporal cues to users with cognitive difficulties.<br><br>It is possible to follow the year's changes in nature, partly with your senses but also through experiences and activities in the outdoor environment.
B9. Symbolism and reflection	There are elements that generate thoughts about the symbol- ism and metaphors existing between one's life and nature. The experience of timelessness in relation to a stone covered with a blanket of moss is one example of such symbolism. However, to some users, nature's power of transformation displayed for instance in intense spring greenery is too overwhelming and even aggressive, since it does not reflect the user's own capacity for transformation.<br><br>There are elements in the outdoor environment that can give rise to symbolism and metaphors between one's own life and nature. The experience of timelessness in the vicinity of a large moss-covered rock is one example.
B10. Space	There are areas offering a restful feeling of entering another world, a coherent whole.<br><br>There are areas that give the feeling of entering an undisturbed world or coherent whole, for example, in a beech forest.
B11. Serene and peaceful (Serene)	There are undisturbed areas that are not crowded. Well- maintained areas and calming elements such as water and greenery offer relaxation, peace and silence. The sounds produced by water are particularly soothing.<br><br>There are peaceful places in the outdoor environment that are neither overpopulated nor have disturbing elements. Well-kept areas with

	soothing elements of water and/or greenery offer relaxation, peace and silence. Pleasant sound of water is especially soothing.
B12. Wildness and nature (Wild nature)	It is possible to experience nature on its own terms. There are areas with plants that seem to be wild and to have developed without human influence.<br><br>There is the opportunity to experience nature on its own terms. There are areas where plants appear to have come by themselves and where they are allowed to develop freely.
B13. Secluded and protected (Refuge)	There are enclosed and secluded, verdant places where users can potter and play, be alone, have private discussions or just sit and watch people from a distance. There are private spaces where staff can take breaks. Some users have a strong need to be alone with nature. To particularly sensitive users, a design with two paths leading to the refuge places is important as it gives the possibility of escape if someone approaches.<br><br>There are surrounded and secluded green places where you can do whatever you want, be left alone, have private conversations or just watch other people from a distance.
